# Current progress on *in vitro* differentiation of ovarian follicles from pluripotent stem cells

**DOI:** 10.3389/fcell.2023.1166351

**Published:** 2023-06-01

**Authors:** Genie Min Ju Wu, Andy Chun Hang Chen, William Shu Biu Yeung, Yin Lau Lee

**Affiliations:** ^1^ Department of Obstetrics and Gynaecology, School of Clinical Medicine, The University of Hong Kong, Hong Kong, China; ^2^ Shenzhen Key Laboratory of Fertility Regulation, Reproductive Medicine Center, The University of Hong Kong—Shenzhen Hospital, Shenzhen, China; ^3^ Centre for Translational Stem Cell Biology, The Hong Kong Science and Technology Park, Hong Kong, China

**Keywords:** primordial germ cells, granulosa cells, follicular development, *in vitro* differentiation, epigenetics, pluripotent stem cells

## Abstract

Mammalian female reproduction requires a functional ovary. Competence of the ovary is determined by the quality of its basic unit–ovarian follicles. A normal follicle consists of an oocyte enclosed within ovarian follicular cells. In humans and mice, the ovarian follicles are formed at the foetal and the early neonatal stage respectively, and their renewal at the adult stage is controversial. Extensive research emerges recently to produce ovarian follicles *in-vitro* from different species. Previous reports demonstrated the differentiation of mouse and human pluripotent stem cells into germline cells, termed primordial germ cell-like cells (PGCLCs). The germ cell-specific gene expressions and epigenetic features including global DNA demethylation and histone modifications of the pluripotent stem cells-derived PGCLCs were extensively characterized. The PGCLCs hold potential for forming ovarian follicles or organoids upon cocultured with ovarian somatic cells. Intriguingly, the oocytes isolated from the organoids could be fertilized *in-vitro*. Based on the knowledge of *in-vivo* derived pre-granulosa cells, the generation of these cells from pluripotent stem cells termed foetal ovarian somatic cell-like cells was also reported recently. Despite successful *in-vitro* folliculogenesis from pluripotent stem cells, the efficiency remains low, mainly due to the lack of information on the interaction between PGCLCs and pre-granulosa cells. The establishment of *in-vitro* pluripotent stem cell-based models paves the way for understanding the critical signalling pathways and molecules during folliculogenesis. This article aims to review the developmental events during *in-vivo* follicular development and discuss the current progress of generation of PGCLCs, pre-granulosa and theca cells *in-vitro*.

## 1 Introduction

Ovary is the fundamental organ supporting female reproductive life. Ovarian function is highly dependent on the number of ovarian follicles, which declines as the women age. In humans, there are around 1–2 million ovarian primordial follicles at birth (Li et al., 2010). The number decreases throughout a woman’s reproductive life, and reaches down to about 1000 by menopause (Hansen et al., 2008; Park et al., 2021). Premature ovarian insufficiency refers to loss of ovarian functions before age of 40 and causes infertility in 7.4% of women (Maclaran and Panay, 2015). Besides infertility, loss of ovarian functions leads to osteoporosis and perimenopausal syndrome, which highly affects the quality of life in some women. To date, an increasing number of young patients are suffering from ovarian dysfunction due to increased incidences of cancer and other potential aetiology [e.g., mental stress (Kaplan and Manuck, 2004) and environmental pollution (Iorio et al., 2014)]. Therefore, preservation of ovarian function becomes an important issue in clinical setting. Currently, hormone replacement therapy is commonly used in perimenopausal women for relieving the menopausal syndrome. However, some evidence has shown that hormone replacement therapy is not suitable for patients with specific health conditions such as history of breast or endometrial cancer (Ross et al., 2000; Narod, 2011). Embryo, oocyte, and ovarian tissue cryopreservation are offered to cancer patients who need chemotherapy or radiotherapy. Nonetheless, the retrieval and preservation of oocytes and ovarian tissues require surgical operations, which pose risks to the women. The limited amount of ovarian tissues and the tissue damages during collection further restrict their treatment efficacy (Sonmezer and Oktay, 2004). Hence, ovarian follicle reconstitution with cells derived from stem cells as an alternative source of ovarian cells has become a hot topic in the field.

Studies have unveiled the ovarian development process in many mammalian species. The initiation of ovarian development involves the formation of bipotential primordial germ cells (PGCs, progenitor cells of oocytes) and pre-granulosa cells (pre-GCs, precursors of granulosa cells). During foetal development, the PGCs undergo a series of decisive events, including lineage specification, migration, proliferation, and sex determination, while the pre-GCs play an essential role in communication with PGCs to form the primordial follicles (Fukuda et al., 2021)**.** The specification and sex determination of the granulosa cells are initially independent **of** the germ cells (Harikae et al., 2013)**.** Several signalling pathways and molecules are involved in the differentiation trajectory of germ cells (Magnúsdóttir et al., 2013; Guo et al., 2015; Otte et al., 2017) and granulosa cells (Stévant et al., 2019; Niu and Spradling, 2020; Fukuda et al., 2021), as well as the formation of follicles (Chen et al., 2014; Zhang et al., 2017). The exact process, however, is not yet clear.

To fully understand the whole process of ovarian follicle formation, *in-vivo* (Barnett et al., 2006; Oktem and Urman, 2010; Abdollahifar et al., 2019) and *in-vitro* (Desai et al., 2010; Skory et al., 2015; Simon et al., 2020) models have been developed to mimic follicular development. These models provide valuable information on developmental events and critical signalling pathways during the process. Moreover, stem cell-derived PGCs and granulosa cells not only provide insights into cell behaviours during folliculogenesis but also pave the way for ovarian follicle reconstitution. Primordial germ cell-like cells (PGCLCs) generated from pluripotent stem cells (PSCs) are well characterized (Hayashi et al., 2011) and shown to have similar behaviours to the embryo-derived PGCs (Ge et al., 2015). Formation of ovarian follicles requires communication between the germ cells and the somatic cells. The production of pre-GCs from PSCs of humans and mice has also been reported recently (Novak et al., 2006; Jung et al., 2017; Wang et al., 2021; Yoshino et al., 2021; Smela et al., 2022). In this review, we first discuss the critical pathways and molecules involved in folliculogenesis including formation of PGCs, pre-GCs and follicles. Next, we review the current understandings of *in-vitro* differentiation of PGCLCs, pre-GCs and theca cells from PSCs. Lastly, we provide our perspective on possible applications of the stem cells-derived products for therapeutic treatments.

## 2 Primordial germ cell development

Embryonic development starts after fertilization. The inner cell mass differentiates into epiblast (EPI) and primitive endoderm (PrE). In mice, the segregation appears in a “salt and pepper” fashion with formation of *Nanog*- and *Gata6*-positive cells, representing the EPI and the PrE cells respectively (Chazaud et al., 2006; Plusa et al., 2008). The PrE mainly contributes to the extraembryonic lineages such as visceral and parietal endoderm. Later, the visceral endoderm further splits into anterior and posterior visceral endoderm. A close interaction of the posterior visceral endoderm and the EPI is crucial for gonadal formation (Stern and Downs, 2012). The secretion of Wnt3 from the visceral endoderm confers competence of germline fate to the EPI, while CER1 (BMP4 inhibitor) and DKK1 (WNT inhibitor) from the anterior visceral endoderm inhibit PGC formation, which together orchestrate PGC formation from the EPI (Paca et al., 2012; Makar and Sasaki, 2020). There are two cell populations in the EPI according to their locations: distal and proximal. It is the proximal EPI that differentiates into mesoderm and PGCs, the founder cells of gametes (Tam and Zhou, 1996; Lu et al., 2001).

**FIGURE 1:**
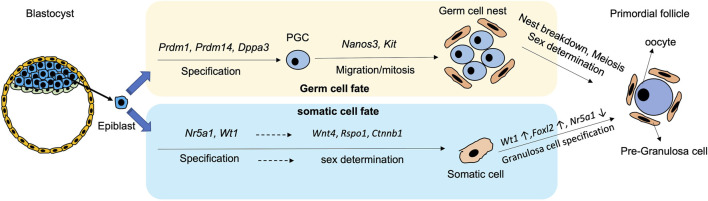
Schematic diagram showing the process and critical genes involve in the derivation of germ cells and pre-granulosa cells from epiblast during differentiation.

PGC specification is modulated by a temporal sequence of gene expressions. In mice, the bone morphogenetic protein (BMP) signalling pathway initially induces *Prdm1* and *Prdm14* at around embryonic day E6.0-6.5 (Figure 1). The cells expressing *Prdm1* and *Prdm14* are restricted to the germ cell lineages, and they suppress the formation of somatic cell lineages (Kurimoto et al., 2008; Yamaji et al., 2008; Updike et al., 2014). PRDM1 induces the expression of *Tcfap2c*, an early germ cell-specific marker, while PRDM14 and TCFAP2C later bound to PGC-specific genes including *Dppa3* (also known as *Stella*)*, Kit,* and *Nanos3* to secure germ cell direction (Magnúsdóttir et al., 2013). In addition, PRDM1 and PRDM14 form a complex with arginine methyltransferase PRMT5 to activate the germ cell programming (Ancelin et al., 2006). Subsequently, *Dppa3* is induced by PRDM14 and expressed at around E7.25 for PGC specification (Figure 1).

After specification, PGCs are driven away from the dorsal hindgut and migrate into the genital ridge for embryonic gonad formation (Molyneaux and Wylie, 2004). During migration, the *Nanos* *3*-positive PGCs undergo a series of mitotic divisions which is known as the proliferation period (Saga, 2008; Suzuki et al., 2008). *Kit* is another germ cell-specific marker from middle to late stage of oocyte development. During migration, KIT interacts with Kit-ligand (KITL) on follicular somatic cells to prevent apoptosis of the PGCs (Godin et al., 1991; Mahakali Zama et al., 2005) (Figure 1). Apart from the PGC-specific genes, mesodermal transcriptional factors such as *Msx1* and *Msx2* moderate PGC migration, as proved by failure of migration of *Msx1*/*Msx2-*null PGCs to the genital ridge and ending up with formation a small population of PGCs (Sun et al., 2016). The migration of PGCs terminates at around E10.5, when the cells reach the genital ridge where they populate by mitosis. At this stage, the PGCs remain bipotential, and are capable of developing into either male or female gametes. Sex determination of the PGCs is initiated only when the PGCs invade into the thickened coelomic epithelium, and form the gonad with their surrounding somatic cells (Piprek, 2010).

The process of human PGC development is mostly conserved as in mice. Human PGCs originate from the wall of yolk sac at around 3 weeks post-fertilization. They undergo migration (fourth week), proliferation (4-11th week) and finally meiosis (around 17th and 19th week in female and male, respectively) (De Felici et al., 2013; Guo et al., 2015). The genes and pathways controlling the PGC development are similar in mice and humans. For example, markers representing early (*PRDM14, DPPA3, KIT, and NANOS3*) and late (*DDX4 and DAZL*) PGC stages in humans are also expressed in mice (Guo et al., 2015)**.** However, slight differences are found between mouse and human PGCs. For instance, the pluripotent gene *Sox2* is expressed in the mouse PGCs (Hayashi et al., 2011) but not in the human PGCs (Guo et al., 2015). Instead, *SOX17* is critical for PGC specification (Irie et al., 2015), while *SOX15* may have a role in maintaining PGC identity in the human PGCs (Guo et al., 2015; Pierson Smela et al., 2019).

Studies demonstrated largely conserved development of germline lineages among mammalian species including livestock (Hyldig et al., 2011; Leitch et al., 2013; Alberio et al., 2021; Soto and Ross, 2021; Chen et al., 2022a). The transcriptome of bovine PGCs at around 50 days of fetal age is essentially similar to that of early human PGCs at mitotic stage with slight differences noted (Soto and Ross, 2021). Bovine PGCs express common germ cell markers such as *PRDM1*, *TFAP2C*, *SOX17* but not *SOX2*, and only a small population of bovine PGCs express *PRDM14*, which is expressed in the human and mouse PGCs (Soto and Ross, 2021). A recent study comparing the transcriptomes of ovarian cells from different mammalian species confirmed the expression of well-recognised PGC markers such as *PRDM1, DDX4* and *DAZL* in porcine and goat PGCs. The study also found that the retinoic acid (RA) signalling pathway-related genes (*STRA8, ZGLP1*) were highly expressed in the human PGCs but not in those of other mammalian species (Chen et al., 2022a).

Global demethylation which erases somatic methylation signatures and parental imprints is crucial for PGC development. It leads to reactivation of genes associated with pluripotency and gametogenesis. At early stage of mouse PGC specification, the repression of *Dnmt1* and *Dnmt3a/b* initiates the first wave of DNA demethylation in PGCs (Kagiwada et al., 2013; Zeng and Chen, 2019). Later, when PGCs migrate into and colonize in genital ridge at around E9.5, the second wave of global DNA demethylation is activated (Hajkova et al., 2002) with activation of *Tet1* and *Tet2* to oxidize 5 mC into 5 hmC (Hackett et al., 2013; Yamaguchi et al., 2013; Piccolo and Fisher, 2014; Clark, 2015). Intriguingly, DNA demethylation regulates the expressions of early germ cell markers like *Prdm1* and *Dppa3* (Mochizuki et al., 2012). Histone modifications in PGCs show a repression in H3K9me2 and a surge in H3K27me3 on E9.5 (Seki et al., 2005; Seki et al., 2007; Saitou et al., 2012). The epigenetic changes in human PGCs are highly similar to those in the mouse PGCs with a few differences; the decrease in H3K9me2 and the increase in H3K27me3 occur before demethylation of the imprinted genes (Eguizabal et al., 2016). These germline-related epigenetic changes are highly conserved in mice and humans (Eguizabal et al., 2016).

## 3 Pre-granulosa cell development

In mice, sex determination of gonads occurs at around E10.5 when *Sry* starts to be expressed. Without the *Sry* expression, the genital ridge is driven to ovarian development (Wilhelm et al., 2013a; Windley and Wilhelm, 2015). The onset of gonadal somatic cell specification initiates at around E9.0-E9.5 with expression of *Nr5a1* (steroidogenic factor1, *SF-1*) and Wilms’ tumor 1 (*Wt1*) (Liu et al., 2016) (Figure 1). *Wt1* plays a critical role in determining the fates of granulosa cells and theca cells. Disruption of *Wt1* in mouse ovary leads to abnormal ovarian structure, where most of the oocytes are surrounded by a single layer of squamous cells without cuboidal granulosa cells (Cen et al., 2020). WT1 also reduces the expression of *Nr5a1* for granulosa cell fate (Chen et al., 2017; Chen et al., 2022b). Later, the expressions of Wnt Family Member 4 *(Wnt4)*, R-spondin1 *(Rspo1)* and β-catenin (*Ctnnb1*) in somatic cells ensure sex determination into the female gonadal cells before further diversion into theca cells and granulosa cells (Pannetier et al., 2016; Li et al., 2017a; Rotgers et al., 2018; Tang et al., 2020).

There are two populations of granulosa cells during mouse ovarian differentiation (Niu and Spradling, 2020). The first population is the bipotential granulosa cells which are characterised by expression of transcriptional factor Forkhead box L2 (*Foxl2*) (Wilhelm et al., 2013b). Although *Foxl2* is not required for initiation of pre-GCs differentiation, it is necessary for their maintenance. A fine-tuned *Foxl2* expression is required for proper formation of the female gonad (Schmidt et al., 2004). Interestingly, the *Foxl2*-expressing cells can give rise to various cell types in the ovary, including granulosa cells, theca-interstitial cells, and ovarian stroma cells depending on lineage-specific gene expressions of these subpopulations (Zhou et al., 2022). The epithelial granulosa cells are the other cell population marked by the expression of Leucine-rich repeat-containing G-protein-coupled receptor 5 (*Lgr5*), a member of the Wnt family (Niu and Spradling, 2020)**.** In mouse ovaries, the *Lgr5-*positive pre-GCs appear at around E14.5, which mainly reside on the ovarian surface. In the adult ovary, these cells are restricted to the ovarian cortex. Intriguingly, the *Lgr5*-positive cells contribute to the second wave of folliculogenesis in postnatal ovary by differentiation into the *Foxl2*-positive cells (Ng et al., 2014; Rastetter et al., 2014; Niu and Spradling, 2019; Fukuda et al., 2021). Therefore, *Foxl2* and *Lgr5* are both important markers of the mous**e** pre-GCs (Wang et al., 2017).

Unlike those in mice, human pre-PC development remains relatively unclear. Several studies on transcriptomes of human ovarian cells provided insights into the differentiation trajectory of these cells. As expected, the human female gonadal somatic cell progenitor cells express *NR5A1*, *WT1,* and *GATA4*, while the granulosa cells express *FOXL2*, similar to those in mice. Differences between the human and the mouse granulosa cell lineages are identified. For example, genes expressed in the mouse pre-GCs (*Wnt6* and *Kitl*) are only expressed in the mature *FOXL2*-positive granulosa cells but not in the pre-GCs of humans (Zhang et al., 2018; Wang et al., 2022). The granulosa cell development of livestock animals is largely unknown. An early study demonstrated importance of activation of Notch and ERK signalling pathways on bovine follicular growth and maturation (Hatzirodos et al., 2014). There are reports showing conservation of granulosa cell development among species. For instance, *Gata4*, an important gene associated with genital ridge formation in mice, is also expressed in porcine early gonadogenesis (McCoard et al., 2001). Similarly, *FOXL2* plays a sex-determining role in goat gonadogenesis, which is supported by a female-to-male reversion in the *FOXL2*-knockout goat embryos (Boulanger et al., 2014). A single-cell RNA-seq analysis revealed expression of granulosa cells markers (*AMHR2*, *WNT6*, and *NR5A1*) in all the studied species despite relatively lower expressions in goats (Chen et al., 2022a). The results supported that the basic regulatory genes during germline development are evolutionarily conserved in mammals.

## 4 Follicular formation and maturation

The final stage of ovarian formation is the assembly of primordial follicles. The breakdown of germ cell cyst inaugurates the process with migration of germ cells from the medullary to the cortical region of the ovary. Concurrently, the *Foxl2*-positive pre-GCs differentiate into granulosa cells in the cortex to support oocyte maturation (Wang et al., 2017). The granulosa cells then migrate, invade, and separate the oocytes (Wear et al., 2016). Each primordial follicle consists of a single oocyte surrounded by a single flattened layer of granulosa cells (Pepling, 2006). Thereafter, the primordial follicles remain inactivated till puberty in humans.

**FIGURE 2:**
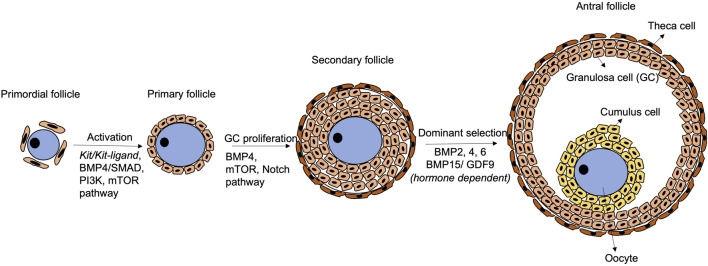
Schematic diagram of follicular development. Primordial follicle activation initiates the transition to primary follicles, with participation of *Kit/Kit-ligand*, BMP4/SMAD, PI3K and mTOR pathways. Later, the BMP4/SMAD, mTOR and Notch pathways regulate granulosa cell proliferation to form secondary follicles. Thereafter, the follicles are gonadotrophin-dependent, and BMP2, 4, 6 and BMP15/GDF9 play crucial roles in modulating the response to FSH and dominant follicle selection. The BMP4/SMAD signalling remains functional during the whole process.

Follicular development initiates with activation of primordial follicles into primary follicles, which involves physical interactions and paracrine communications via critical signaling pathways between the oocyte and the granulosa cells (Figure 2). In mice, KIT (in oocytes)/KITL (granulosa cells) communication promotes follicular activation and primordial-to-primary follicle transition. During mouse ovarian tissue culture, treatment with antibody against KIT retards the transition, and the effect can be rescued by exogenous KITL treatment (Parrott and Skinner, 1999). Besides, the phosphatidylinositol 3-kinase (PI3K) pathway in mouse growing oocytes affects follicular activation (Reddy et al., 2005). *Akt* and *Foxo3* serve as down-stream genes of the PI3K pathway controlling the primordial follicle pool in a positive and negative manner, respectively (Li et al., 2021a). Interestingly, the KIT*/*KITL signalling activates the PI3K pathway to mediate folliculogenesis (Kissel et al., 2000). In fact, the PI3K pathway may be the primary down-stream mediator of KIT*/*KITL from the primordial follicle formation stage (Burton et al., 2022).

Mammalian target of rapamycin (mTOR) pathway and Notch pathway are also crucial to follicular development in mice and humans (Figure 2). In mice, inhibition of mTOR leads to failure of granulosa cells penetration between oocytes and nest breakdown (Yu et al., 2011; Cheng et al., 2015). In humans, the mTOR pathway is related to ovarian diseases such as polycystic ovarian syndrome, which exhibits insulin resistance due to excessive stimulation of the mTOR pathway (Liu et al., 2018)**.** However, the connections between mTOR and the disease aetiology are yet to be fully revealed (Liu et al., 2018). Notch (somatic-cells)/Notch ligand (oocyte) communication is also critical for follicular formation in mice and humans. Granulosa cell proliferation is inhibited when the Notch pathway is inactivated (Zhang et al., 2011), resulting in abnormal follicles with larger size oocytes surrounded by gradually less granulosa cells (Vanorny et al., 2014).

Fine-tuned expressions of genes of the BMP family together with *Gdf9* (growth differentiation factor 9), a member of the TGF-β family, are required during the whole folliculogenesis process in mice and humans (Wu and Matzuk, 2002; Mottershead et al., 2012; Belli et al., 2018). During follicle activation, the BMP4/SMAD signalling not only promotes primordial-to-primary follicle transition, but also ensures viability of the developing follicles by their synergistic effects (Ding et al., 2013; Rossi et al., 2016). Co-expression of *BMP15* and *GDF9* ensures communication between the oocyte and the granulosa cells (Otsuka et al., 2001). In the late follicular development, *BMP15* and *GDF9* also control cumulus cell expansion and apoptosis to maintain the stability of follicles during their growth (Gode et al., 2011; Paulini and Melo, 2011; Sanfins et al., 2018).

Folliculogenesis is gonadotrophins dependent starting from the secondary follicle stage. A secondary follicle, known as antral follicle, consists of an antrum filled with follicular fluid. The follicular fluid contains hormones, proteins and other factors derived from bloodstream and granulosa cells. FSH supports growth of the antral follicles and stimulates the granulosa cells to produce oestrogen which suppresses the FSH effect. The follicle, that is, most responsive to FSH continues to grow and becomes dominant, while the less responsive follicles undergo atresia (Scaramuzzi and Webb, 1995; Baerwald et al., 2012; Combelles, 2013; Telfer and Andersen, 2021).

## 5 Germline stem cells

There are reports on isolation of adult stem cells from mouse or human ovarian tissue, termed female germline stem cells (Hong-Thuy et al., 2023; Wang et al., 2023) or oogonial stem cells (Grieve et al., 2015). The concept of oogonial stem cells was first brought up by Tilly and co-workers in 2004 after showing the existence of proliferative cells in postnatal ovary that could sustain the production of ovarian follicles (Johnson et al., 2004). Similar cell populations were isolated in other species, such as chickens (Meng et al., 2023), bovines (Grieve, 2017) and humans (Clarkson et al., 2018; Silvestris et al., 2018). The reported stem cells were mainly localized to the cortical area of ovary and were isolated by the surface marker, DDX4. These cells expressed *Oct4, Dppa3, Prdm1,* and *Dazl* (Ding et al., 2016). Intriguingly, *DDX4*-positive cells from ovarian tissue of menopausal women contributed to formation of *GDF9* (oocyte marker) and *SYCP3* (meiosis marker) expressing oocyte-like cells after 3 weeks of culture (Silvestris et al., 2018). Moreover, another study demonstrated that ovarian injury triggered tissue repair and regeneration through the reactivation, proliferation and differentiation of BrdU^+^/*DDX4* ^+^ stem cells (Erler et al., 2017).

Despite the positive findings in some reports, the existence and function of oogonial stem cells are controversial (Yoshihara et al., 2023). Early in 2015, a study failed to find contribution of isolated *DDX4*-positive cells from mouse and human ovaries to oocytes after their injection into ovarian tissue (Zhang et al., 2015). The identity of the isolated “oogonial stem cells” with positive *Ddx4* but negative *Dazl* and *Dppa3* expressions was further criticized in another study (Zarate-Garcia et al., 2016). More recently, a single cell RNA-sequencing study on human ovarian tissue revealed that *DDX4*-positive cells were likely to be perivascular cells but not oogonial stem cells (Wagner et al., 2020). Hence, the controversy deters the use of the *DDX4*-positive cells for regeneration of ovarian tissue in postnatal life.

## 6 Current progress on *in-vitro* differentiation of PGCs

### 6.1 PGCLC induction from pluripotent stem cells

Different types of mouse PSCs (mPSCs) are excellent cell models for deriving PGCs *in-vitro* and functional study *in-vivo*. Common PSCs include mouse embryonic stem cells (mESCs) which are originated from the inner cell mass of blastocysts (Young Richard, 2011) and iPSCs which are reprogrammed from somatic cells to acquire pluripotency (Takahashi and Yamanaka, 2006). Ground-state mPSCs cultured in mitogen-activated protein kinase inhibitor PD0325901, glycogen synthase kinase inhibitor CHIR99021 and leukemia inhibitory factor (2i/LIF) are pluripotent and contribute to all embryonic lineages in foetuses (Nichols et al., 2009; Young Richard, 2011) including germline (Nichols et al., 2009; Tian et al., 2021). Expanded potential stem cells (EPSCs) is a new type of stem cells with both embryonic and extraembryonic differentiation potential (Yang et al., 2017). Using a cocktail of inhibitors targeting pathways involved in blastomere differentiation such as MAPKs, Src and Wnt/Hippo/TNKS1/2, mESCs can be converted into mEPSCs with expanded pluripotency (Yang et al., 2017). Both PSCs and EPSCs contribute to gonadal formation and germline-transmission in chimera *in-vivo* (Nichols et al., 2009; Yang et al., 2017).

To differentiate mouse PGCs from PSCs *in-vitro*, critical molecules and signalling pathways are identified from *in-vivo* studies. BMP signalling pathway is first identified from genetic studies showing that extraembryonic mesoderm-secreted BMP4 is required for mouse PGC generation (Lawson et al., 1999). Strong expression of *Bmp4* in inner cell mass is observed from E3.5 till E7.5 when EPI differentiates terminally into PGCs in mice (Dudley et al., 2010). The importance of BMP signalling is confirmed in the *Bmp4*-and *Bmp8b*-null mouse models, the EPI of which cannot contribute to the formation of PGCs, but the phenotypes can be rescued by exogenous BMP8b protein treatment (Ying et al., 2001). The SMAD pathway, which is downstream of BMP signalling, also plays an important role in PGC formation; *Smad*1/5/8 are activated in migration of PGCs and maintenance of germline competence of PGCs (Chang and Matzuk, 2001; Hayashi et al., 2002; Whyte et al., 2015). Another major pathway in PGC differentiation is the Wingless/INT-1 (Wnt) pathway (Tanaka et al., 2013; Cantú et al., 2016). During PGC specification and meiosis activation, the Wnt signalling is downregulated via degradation of β-catenin, a key regulator of the Wnt pathway, whereas the Wnt pathway is upregulated during PGC mitosis (proliferation) (Kimura et al., 2006; Lee et al., 2016; Le Rolle et al., 2021; Morgani and Hadjantonakis, 2021).

**FIGURE 3:**
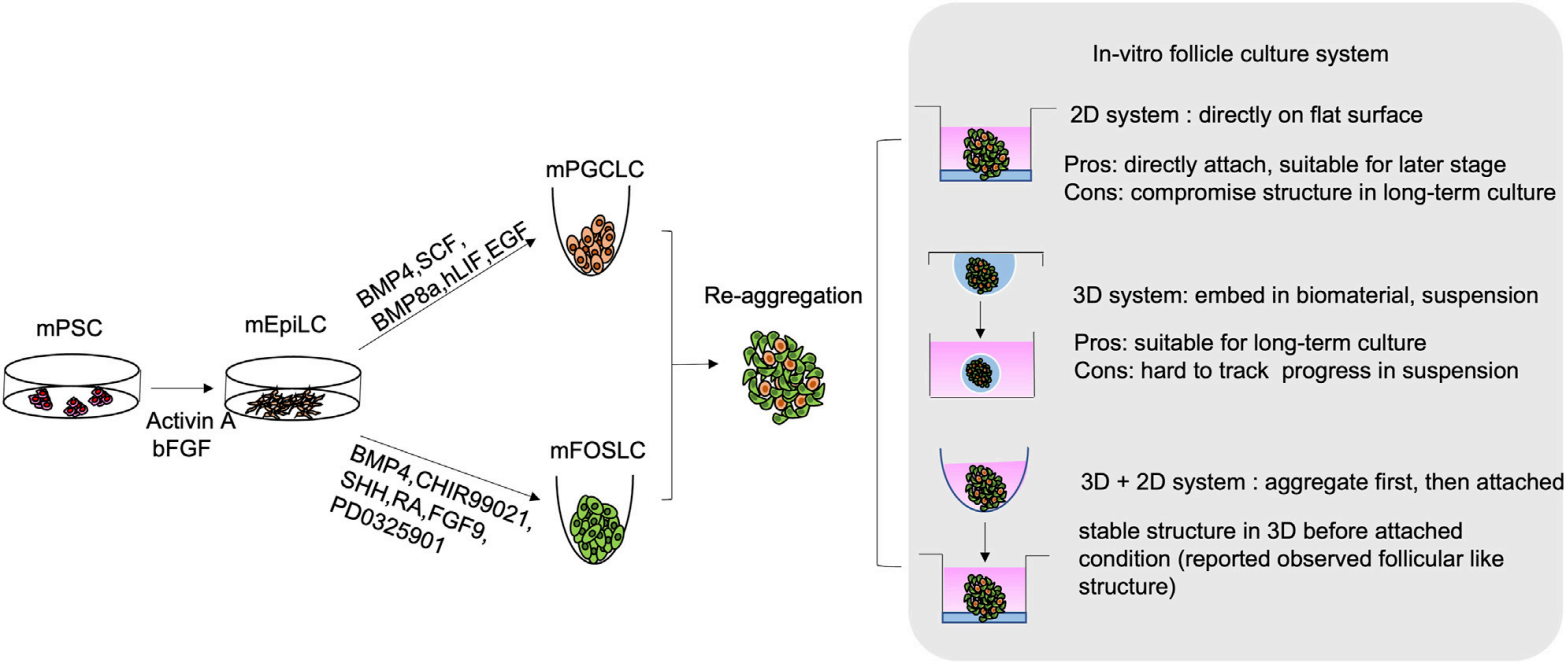
Schematic diagram of *in-vitro* generation of mouse follicular like structure from mouse pluripotent stem cell (mPSCs). mPSCs are driven to mouse epiblast-like cells (mEpiLCs) in the presence of Activin A and bFGF. mEpiLCs are further differentiated into mouse primordial germ cell-like cells (mPGCLCs) and mouse fetal ovarian somatic cell-like cells (mFOSLCs) with different conditions. After isolation of mPGCLCs and mFOSLCs, the two population are reaggregated for subsequent follicular culture. 2D and 3D culture system as well as mixed approach are reported as listed.

By modulating the critical signalling pathways involved in PGC specification, successful derivation of PGCs from mESCs was first reported in 2011. In the protocol, mESCs were first differentiated into EPI-like cells (EpiLCs), a critical stage for mouse PGC development *in-vivo*. To achieve this, basic fibroblast growth factor (bFGF) and activin A were added to inhibit the Wnt pathway and activate the SMAD pathway. The EpiLCs were further differentiated into PGCLCs by BMP4, BMP8a, stem cell factor (SCF), hLIF and epidermal growth factor (EGF) (Hayashi et al., 2011) (Figure 3). The PGCLCs not only showed germline properties with expressions of germ cell markers (*Prdm1, Dppa3, and Nanos3*), but also showed global DNA demethylation, accompanied with decrease in *Dnmt3a/b* expressions. These characteristics correspond to the those of the PGCs *in-vivo*. Moreover, germline competence of the ESC-derived PGCLCs was illustrated by the formation of follicular-like structure upon aggregation and co-culture with gonadal somatic cells (Hayashi et al., 2012). Critically, the oocytes isolated from the aggregates can give rise to offspring after *in-vitro* fertilisation (Hayashi et al., 2017), which is the golden standard for examining the developmental competence of PGCLCs. Similarly, miPSCs were later reported to contribute to PGCLCs by adapting the same protocol (Hayashi and Saitou, 2014).

The generation of human PGCLCs is achieved using similar approaches as for the mouse PGCLCs (Sasaki et al., 2015; Sugawa et al., 2015; Irie et al., 2017; Murase et al., 2020a). The major components used for mouse and human PGCLC differentiation are listed in Table 1. Due to different pluripotent states of the mouse and human PSCs, their PGCLC differentiation protocols are slightly different. The primed human ESCs (hESCs) share similar characteristics as the mouse EpiLCs (Tesar et al., 2007). Therefore, human PGCLC differentiation is initiated by first converting the hESCs to mesoderm-like cells in the presence of activin A and Rho-associated protein kinase (ROCK) inhibitor. BMP4 is then supplemented for induction of BMP signaling in both the mouse and human differentiation protocols. BMP2 has also been used in the human PGCLC differentiation (Irie et al., 2015; Kobayashi et al., 2017; Jostes and Schorle, 2018; Gao et al., 2019). In fact, both BMP2 and BPM4 are secreted by the extraembryonic mesoderm to support the development of human PGCs *in-vivo* (Irie et al., 2015; Jostes and Schorle, 2018). To date, the differentiation and competence of mouse and human PGCLCs from PSCs have been extensively reported. However, there are still missing pieces of the puzzle, for example, the factors affecting maturation of these PGCLCs into oocyte-like cells are unknown. Hence, future research such as tracking the developmental progress during PGCLCs maturation is needed to provide insights into the critical genes and pathways for further optimisation of the *in-vitro* protocol.

**TABLE 1:** The cell sources, intermediate cell stages, main components for inducing the intermediate stages and PGCLCs from mouse and human cells.

Species	Mouse	Human
Cell sources	ESCs Hayashi et al. (2011), Ohta et al. (2017), iPSCs Hayashi and Saitou, (2014), adult stem cells Ge et al. (2016); Tan et al. (2016)	ESCs, iPSCs Sasaki et al. (2015), Sugawa et al. (2015), Murase et al. (2020a), EPSCs Gao et al. (2019), PSCs Kobayashi et al. (2017)
Intermediate stage	EpiLCs Hayashi et al. (2011)	Pre-mesendoderm Kobayashi et al. (2017), mesoderm-like cells Zhang et al. (2021b)
Main components for intermediate stage induction	Activin A Hayashi et al. (2011), bFGF Hayashi et al. (2011)	Activin A Sasaki et al. (2015), Sugawa et al. (2015), Murase et al. (2020a), CHIR99021 Sasaki et al. (2015), Sugawa et al. (2015); Murase et al. (2020a), bFGF Sugawa et al. (2015), ROCK inhibitor Sasaki et al. (2015), Sugawa et al. (2015), Murase et al. (2020a)
Main components for PGCLC induction	BMP4 Hayashi et al. (2011), Ohta et al. (2017), hLIF Hayashi et al. (2011), Ohta et al. (2017), SCF Hayashi et al. (2011), Ohta et al. (2017), BMP8b Hayashi et al. (2011), EGF Hayashi et al. (2011); Ohta et al. (2017)	BMP4 Sasaki et al. (2015), Sugawa et al. (2015), Kobayashi et al. (2017), Gao et al. (2019), Murase et al. (2020), BMP2 Irie et al. (2015), Kobayashi et al. (2017), Jostes and Schorle (2018), Gao et al. (2019), hLIF Sasaki et al. (2015), Sugawa et al. (2015), Kobayashi et al. (2017), Gao et al. (2019), Murase et al. (2020), SCF Sasaki et al. (2015); Kobayashi et al. (2017), Gao et al. (2019), Murase et al. (2020), EGF Sasaki et al. (2015), Murase et al. (2020), ROCK inhibitor Sugawa et al. (2015), Kobayashi et al. (2017), Gao et al. (2019)

### 6.2 Epigenetic regulation of PGCLC induction

In addition to germline markers, the expressions of epigenetic regulators are studied in the PSC-derived PGCLCs. Similar to the *in-vivo* patterns, the expressions of *Dnmts* are decreased along the differentiation of mPGCLCs (Hayashi et al., 2011). Published chromatin immunoprecipitation (ChIP)-sequencing datasets demonstrated a decrease in H3K9me2/H3K9me3 and an increase in H3K27ac/H3K4me3 during transition from mEpiLCs to mPGCLCs, consistent with the *in-vivo* patterns (Liu et al., 2014; Kurimoto et al., 2015; von Meyenn et al., 2016). Despite the similarity of epigenetic behaviours between mouse and human PGCs, slight differences between the two species were reported. A study demonstrated the DNA demethylation rate of *in-vitro* derived human PGCLCs was much slower than that of mouse PGCLCs (methylation rate at 40% on day four differentiation of mouse PGCLCs *versus* 68% in human PGCLCs). The expression patterns of DNA methylation related genes, *Dnmts* and *Tet* family also showed diversities among the two species. Strong reduction of *Dnmts* expression in the mouse PGCLCs was not seen in the humans, likewise, upregulation of *Tet* was only seen in the mouse PGCLCs but not in the humans (von Meyenn et al., 2016).

### 6.3 PGCLC expansion

The efficiency of the established protocols for PGCLC differentiation from mESCs, miPSCs and hESCs (around 8%–20%) remain low (Hayashi and Saitou, 2013; Irie et al., 2017)**.** The low number of PGCLCs formed *in-vitro* limits its utilisation for drug screening or future therapeutic applications. Therefore, several approaches were taken to expand mouse and human PGCLCs *in-vitro* (Table 2). The mESC-derived PGCLCs was developed by culturing the cells on the m220-5 feeder, a kind of mouse embryonic fibroblast feeder secreting stem cell factor (SCF), which supports the growth of the PGCLCs (Ohta et al., 2017). Chemical screening studies identified candidates, including Rolipram, the phospho-di-esterase 4 (PDE4) inhibitors and forskolin, that targeted the cAMP signalling to enhance the genesis and proliferation of mouse PGCLCs, (Koshimizu et al., 1995; Morita and Tilly, 1999; Pierre et al., 2009; Keravis and Lugnier, 2012; Tan et al., 2016; Ohta et al., 2017; Endo et al., 2019)**.** Upon a 7-day cell expansion with Rolipram and forskolin treatments, the number of PGCLCs was increased by 50-fold, and the transcriptomes and epigenetic signatures of PGCLCs were essentially maintained, despite changes in some PGCLC marker genes (*Ddx4, Dazl* and *Stra8*) and DNA-methylation related genes (*Dnmt3a/3b*) (Ohta et al., 2017; Ishikura et al., 2022). Transcriptome analysis revealed close clustering of the PGCLCs before and after extended culture with *in-vivo* isolated PGCs from E9.5 to E11.5. The PGCLCs after extended culture showed a more comprehensive erasure of DNA methylation similar to that in PGCs at around E13.5. More importantly, the expanded PGCLCs demonstrated competency for spermatogenesis upon injection into neonatal testes (Nagaoka et al., 2020) and competency of oogenic fate with *Zglp1* and *Stra8* expressions upon BMP2 and RA treatments (Nagaoka et al., 2020).

**TABLE 2:** Current approaches of pre-granulosa cell derivation and critical components.

Approaches	Starting cells	Components
Spontaneous differentiation using embryo body	mESCs Novak et al. (2006)	Complete ESC medium without hLIF
Signalling pathway modulators	mESCs Wang et al. (2021), Yoshino et al. (2021)	BMP4, CHIR99021
		→ SHH, RA, PD0325901
		→ BMP4, FGF9 Yoshino et al. (2021)
		Promotor: AM580, Vitamin C Wang et al. (2021)
Transcriptional Regulation	hiPSCs, hESCs Jung et al. (2017), Smela et al. (2022)	Overexpression of NR5A1 Smela et al. (2022), RNUX1 Smela et al. (2022), RUNX2 Smela et al. (2022), *DAZL* Jung et al. (2017), and *BOULE* Jung et al. (2017)

The molecules that work for the mouse PGCLC expansion, including forskolin, rolipram and SCF, also facilitate the expansion of human PGCLCs cultured with STO feeder cells (Gell et al., 2020). The human PGCLC identity is maintained during the expansion as demonstrated by co-expressions of *SOX17, TFAP2C,* and *PRDM1* (Gell et al., 2020) and modest global DNA demethylation (Gell et al., 2020). Moreover, the level of H3K27me3 is increased in the expanded hPGCLCs, similar to that in hPGCLCs before expansion (Sasaki et al., 2015). The culture system can support the expansion growth and epigenetic characteristics of hPGCLCs up to 4 months in culture (Murase et al., 2020). Importantly, the expanded hPGCLCs can form *DDX4-*and *DAZL-*positive oogonia when co-culture with mouse E12.5 gonadal somatic cells (Murase et al., 2020; Mishra et al., 2021). A feeder free expansion culture system for hPGCLCs was reported recently. In this system, the hPGCLCs were directly seeded on Matrigel-coated surface and cultured in SCF, EGF and hLIF supplemented STO feeder-conditioned which suppresses expression of pluripotency-associated markers (such as *SOX2*), and the hPGCLCs can be cultured for 70–153 days (Kobayashi et al., 2022). The resulting expanded PGCLCs exhibited global DNA demethylation, increase in H3K27me3 and decrease in H3K9me2 levels. More importantly, they were competent of forming *DAZL*-positive cells resembling M-prospermatogonia in a reconstituted testis organ culture system (Kobayashi et al., 2022). Based on the above studies, the development of hPGCLC expansion system especially in feeder free condition may provide a strong tool for future application such as drug screening.

Despite the exciting results of the PGCLC expansion as mentioned above, the method was not utilized in most of the follicular reconstitution models. The possible reason may be, in mouse, the source of m220-5 feeders is an obstacle for expansion because it requires genetic modification on mouse embryonic fibroblast feeder (Majumdar et al., 1994). Feederfree expansion system needs to be developed in future. For human, in spite of the reported long-term culture, the competency of the expanded cells was only done on testis reconstitution. The competence of the expanded PGCLCs in ovarian follicle reconstitution still requires further studies.

### 6.4 PGCLC models in livestock animals

PGCLC generation from livestock such as porcine and cattle were reported. Among them, differentiation of porcine PGCLCs from iPSCs is relatively well-studied (Dyce et al., 2006; Wang et al., 2016; Gao et al., 2019). Early in 2006, porcine skin-derived adult stem cells were first used for PGCLC differentiation. After culture of embryoid bodies (EB) from skin-derived stem cells in medium containing follicular fluid, a cell subpopulation expressing the late germ cell markers, *DAZL* and *DDX4*, was found (Dyce et al., 2006)*.* Later, differentiation systems without *in-vivo* derived components were described (Wang et al., 2016; Pieri et al., 2022). Similar to that of mouse, porcine iPSCs are induced to EpiLCs before further differentiation to PGCLCs. The stimulants for the inductions are mostly identical: activin A and bFGF for EpiLC induction, and BMP4, BMP8B, SCF, hLIF and EGF for PGCLC induction (Wang et al., 2016). The porcine PGCLCs express germ cell markers like *PRDM1, PRDM14, DPPA3, DAZL,* and *DDX4* as in the mouse PGCLCs. Although the developmental trajectory of the porcine PGCLCs mainly follows that of mouse, the induction of *SOX17* expression found in porcine PGCLCs also indicates the similarity to that of human (Wang et al., 2016). Several studies reported the expansion protocol for porcine PGCLCs (Yan et al., 2019; Zhang et al., 2021a). The porcine PGCLC proliferation can be promoted by RA, which increases the proportion of cells in the S and G2/M phase but reduce those in the G0/G1 phase (Yan et al., 2019). Luteinising hormone (LH) can enhance PGCLC proliferation by increasing the expression of proliferation-related genes (*CDK1, CDK2*) and reducing that of the apoptosis genes (*P21*, *P53*). It is also shown that LH promotes the expansion during differentiation through the Hippo pathway (Zhang et al., 2021a).

Bovine PGCLCs differentiated from PSCs were also reported (Malaver-Ortega et al., 2016; de Souza et al., 2017). In the presence of BMP4 and RA, *DDX4* expression was detected in the EB derived from bovine iPSCs (Malaver-Ortega et al., 2016). Bovine ovarian stem cells were reported to be a source for PGCLC derivation upon treatments with BMP2, BMP4 or follicular fluid (de Souza et al., 2017). Goat PGCLCs have been derived from EB of iPSCs in the presence of BMP4 and RA (Singhal et al., 2015). The expressions of germ cell markers including *DAZL*, *DPPA3,* and *DDX4* were initiated upon 10 days of culture and maintained till around day 30. With further culture, the detection of oocyte marker *GDF9* in the EB suggested formation of oocyte-like cells (Singhal et al., 2015). Using similar PGCLC differentiation strategy and extended culture, oocyte-like cells surrounded by cumulus cells were observed in goat ESC-derived PGCLCs (Malik et al., 2020). Importantly, the oocyte-like cells can be parthenogenetically activated and form blastocyst-like structure (Malik et al., 2020). Apart from chemical induction of PGCLCs, overexpression or ectopic expression of critical genes during germ cell production was reported in goat adult stem cells (Li et al., 2017b). Later, co-transfection of mRNA of *STRA8, BOULE*, and *DAZL* into goat mesenchymal stem cells resulted in high expression of germ cells markers (*DPPA3, KIT*) and meiosis markers (*SCP3*), providing a new approach to derive goat PGCLCs (Zhang et al., 2019). The understanding of PGCLC development in livestock is important for efficient production of high-quality livestock and preservation of valuable and endangered breed for future generations.

## 7 Current progress on *in-vitro* differentiation of pre-granulosa cells

The concept of ovarian reconstitution for therapeutic treatment of infertility has been brought up for decades. To achieve reconstitution, both germ cells and ovarian somatic cells are required for follicular formation. While PGCLC derivation and expansion have been reported to produce oocyte progenitors *in-vitro*, the source of the supporting somatic cells is limited, hence underlining the necessity of generating follicular granulosa cells *in-vitro*.

The biology of granulosa cells development is largely unknown. To date, only very few studies reported successful generation of granulosa cells *in-vitro* (Table 3). An early study observed follicle-like structures after culture of EB for 18–20 days in complete ESC medium without hLIF (Novak et al., 2006). Estrogen production was detected after 11–20-day of culture, suggesting the formation of functional follicular somatic cells. However, abnormal expressions of meiosis markers were observed in these somatic cells (Novak et al., 2006).

**TABLE 3:** Summary of recent studies on expansion of mouse and human PGCLCs.

Study	Species	Culture conditions
Ohta et al. (2017)	Mouse	Feeder: m220, basal medium: GMEM
		Components: Rolipram, RA, Forskolin, SCF
Murase et al. (2020)	Human	Feeder: m220, basal medium: DMEM
		Components: Forskolin, SCF, bFGF
Gell et al. (2020)	Human	Feeder: STO, basal medium: GMEM
		7F medium: SCF, stromal cell-derived factor 1, bFGF, BMP4, hLIF, N-acetylcysteine, Forskolin
		FR10 medium: Rolipram, Forskolin, SCF
Ishikura et al. (2021)	Mouse	Feeder: m220-5, basal medium: GMEM
		Components: Rolipram, Forskolin, SCF, cyclosporin A
Kobayashi et al. (2022)	Human	Initial expansion phase
		Feeder: STO, basal medium: GMEM
		Components: LIF, EGF, and SCF
		Long-term culture (feeder-free)
		Matrigel-coated surface, basal medium: STO-conditioned medium
		Components: SCF, EGF, and LIF (serum-free)

Despite the above observation, generation of gonadal somatic cells with proper functions is still challenging. The interaction between PGCs and surrounding granulosa cells highly affects the growth and differentiation of both cell types. To better understand the time-dependent interaction among follicular cells, single cell RNA-sequencing was used to elucidate the trajectory and crucial signalling pathways for pre-GC differentiation **in** mouse and human ovarian cells (Stévant et al., 2019; Niu and Spradling, 2020; Wang et al., 2020; Fukuda et al., 2021; Chen and Gao, 2022; Garcia-Alonso et al., 2022). Not until recently, by following the developmental trajectory, the protocol for deriving foetal ovarian somatic cell-like cells (FOSLCs) from mESCs was reported (Figure 3). Similar to PGCLC differentiation, the protocol for FOSLC differentiation also starts with the differentiation of EpiLCs from mESCs. The next step involves addition of BMP4 and CHIR99021 to induce the formation of nascent mesoderm, which is associated with expression of platelet-derived growth factor receptor-a (*Pdgfra*). Subsequently, sonic hedgehog (SHH), RA and FGF inhibitor PD0325901 are added to promote the expression of *Gata4*, a primitive marker of genital ridge formation. Finally, BMP4 and FGF9 are supplemented to the culture to stimulate FOSLC formation with *Nr5a1* and *Foxl2* expressions. The competence of the resulting FOSLCs was demonstrated by follicular-like structures formation after aggregation with PGCLCs (Yoshino et al., 2021).

Soon after, another study reported the derivation of *Gata4-*and *Foxl2-*double positive pre-GCs from mESCs using a 2-dimensional culture system. Their approach aimed at activating Wnt, RA and Hippo pathways which are highly expressed in E12.5 and E13.5 mouse gonadal somatic cells (Wang et al., 2021). By chemical screening, it was found that **a** combination of AM580 and Vitamin C induced the expression of *Foxl2* and *Gata4* and activated the RA and Wnt-related pathways in the treated cells. The *Foxl2*-positive cells, termed “E12.5 gonadal somatic cell like cells (E12.5 GSCLCs)” could activate meiosis of the *in-vivo* derived mPGCs (Wang et al., 2021).

More recently, a robust protocol for production of mouse somatic progenitor cells was also reported. After treating the epiblast-like cell/epiblast stem cells (EpiLCs/EpiSCs) with bFGF, BMP4, RA, Activin A, and CHIR99021, a cell population co-expressing *Gata4* and *Wt1* was obtained. These cells exhibited transcriptomes similar to that of E11.5 mouse gonadal somatic cells. Unfortunately, they only contributed to *Sox9*- (pre-testis somatic cells) but not *Nr5a1*-positive cells (pre-granulosa cell progenitor) upon further differentiation in the presence of bFGF, BMP4, RA, Activin A and Wnt agonist, suggesting that these *Gata4*/*Wt1-*positive cells can only be viewed as gonadal somatic progenitor cells (Gonen et al., 2022).

In addition to modulation of signalling pathways using recombinant proteins and chemicals as in mice, modulation of critical transcription factors was used in human pre-GC differentiation. Early approach was to overexpress critical germ-cell genes in hESCs. For examples, overexpression of *DAZL*, a late germ cells marker, and *BOULE*, a germ cell meiotic marker, in hESCs produces *DDX4-* (late germ cell marker) and *SYCP3-* (germ meiotic marker) double positive cells, which form follicle-like structures when culture with GDF9 and BMP15 (Jung et al., 2017). A recent study also used transcriptional factor library to screen critical factors for deriving granulosa cells from hiPSCs, and *NR5A1*, *RNUX1* or *RUNX2* were identified as critical factors in granulosa cell fate decision (Smela et al., 2022). The differentiated granulosa cell-like cells expressed granulosa cell markers including *AMHR2*, *CD82*, *FOXL2*, *FSHR*, *IGFBP7*, *KRT19*, *STAR*, and *WNT4*, and responded to exogenous FSH by producing oestrogen and progesterone (Smela et al., 2022). However, the developmental stage of the *in-vitro* differentiated pre-GCs is unknown (Jung et al., 2017). Lack of evidence showing pre-GCs to support prolonged culture of PGCLCs, suggesting that the protocol requires further optimization. The approach provides an efficient way to produce human granulosa cell-like cells and paves the way to establish *in-vitro* human ovarian organoid as model for studying interaction between follicular cells.

## 8 Current progress on *in-vitro* differentiation of theca cells

Theca cells are endocrine cells that appear only after formation of secondary follicles, which are characterised by having an antrum, a granulosa cell layer covered by a basal lamina with a theca cell layer on the outside of the follicle. The secondary follicles are generally enclosed by 3–5 layers of theca cells known as theca interna. Meanwhile, steroidogenesis of the theca interna plays a critical role in follicular maturation and ovulation. The outermost layer of the follicle is the theca externa serving as connective cells for structural support of follicles (Magoffin, 2005; Liu et al., 2015; Liu et al., 2020; Vlieghe et al., 2023). Till now, the mechanism of theca cell development is still unclear, though it is shown that the granulosa cells stimulate the function and differentiation of the theca cells (Tajima et al., 2006; Liu et al., 2015) and that the interactions between the oocyte and the granulosa cells highly affect lineage specification of theca cells (Liu et al., 2015).

Several pathways are critical for theca cell differentiation. Oocyte-related molecules, GDF-9/BMP15, promote proliferation and maturation of the theca cells (Vitt et al., 2000; Chang et al., 2017). The Hedgehog pathway is also believed to modulate the growth of theca cells. *PTCH1/2* of the hedgehog pathway are expressed in both human and mouse theca cells and are involved in regulating the differentiation of theca cells (Liu et al., 2020). Until now, there is no report demonstrating successful generation of theca cells from PSCs. However, several studies described the generation of theca cells from mouse and human adult theca stem cells (Honda et al., 2007; Dalman et al., 2018). The mouse theca stem cells can be isolated from ovarian tissues and cultured in a serum-free germline stem cells medium containing EGF, bFGF, hLIF and recombinant rat glial cell line-derived neurotrophic factor (GNDF) (Honda et al., 2007). Serum supplementation induces differentiation of the theca stem cells into LH-receptor expressing steroidogenic cells, to secrete androstenedione and to possess typical morphology of steroidogenic cells with accumulation of lipid droplets and formation of smooth ER (Honda et al., 2007). Likewise, human theca stem cells can be isolated and cultured in medium supplemented with EGF, bFGF, hLIF, GDNF, LH, insulin-like growth factor-1(IGF1) and SCF. After 12 days of culture, a subpopulation of cells with lipid droplets in cytoplasm and expressions of *LHR, GLI2, GLI3, PTCH1, CYP17a1,* and *FSHR* indicating human theca progenitor cell lineage is detected (Dalman et al., 2018). The cells secrete oestrogen into medium (Dalman et al., 2018). These studies provide insights into the essential pathways and factors required for theca cell specific differentiation protocol from PSCs.

## 9 Current progress on *in-vitro* reconstitution of ovarian follicles

Ovarian follicle culture has been developed since late 20th century. Till now, culture systems for different follicular stages have been developed in several species (Hartshorne, 1997; Simon et al., 2020) including two-dimensional (2D) culture systems for bovine preantral follicles (Araújo et al., 2014) and culture system for development of porcine primordial and primary follicles to mature meiotic oocytes with fertilization ability (Telfer et al., 2000; Kaneko et al., 2003). By overexpression of *DAZL* and *BOULE* with treatments of recombinant human GDF9 and BMP15, human follicle-like structures with oocyte-like cell of unknown developmental competence can be produced from hESC-derived EB with variable efficiencies (Jung et al., 2017). Excitingly, functional ovarian reconstitution in mice by co-culture of PGCs with somatic cells has been achieved. The *in-vitro* culture of mouse reconstituted ovary comprising mESC-derived PGCLCs and E12.5 mouse gonadal somatic cells was reported in 2012. The artificial oocytes could be used to produce live pups (Hayashi et al., 2012). Using a similar approach, human PGCLCs in aggregate with fetal ovarian somatic cells underwent a series of meiotic progression into oocytes (Yang et al., 2022). More recently, artificial follicles generated solely by mESC-derived PGCLCs and FOSLCs was reported. In the report, *Prdm1* or *Dppa3* expressing PGCLCs were aggregated with either *Nr5a1-*or *Foxl2*-positive pre-GCs, representing E9.5 PGC and E12.5 pre-granulosa cells *in-vivo* respectively (Hayashi et al., 2012; Yoshino et al., 2021). During the first week of aggregation, the PGCLCs matured and underwent meiosis with expression of *Sycp3*. From the second to third week of culture, reallocation of cells and follicular structures were observed (Yoshino et al., 2021). The study was the first to demonstrate the possibility of *in-vitro* follicular generation.

The *in-vitro* culture conditions are crucial for successful follicular reconstitution. Both 2D and 3D systems are reported for ovarian follicle culture (Figure 3). The 2D system adopted from ovarian tissue culture was predominantly used for follicle culture (Telfer et al., 2008; Motamed et al., 2017). It involves direct seeding of the isolated follicles onto a flat surface (culture well/dish or membrane) with or without extracellular matrix (ECM) such as collagen. However, long-term 2D culture may lead to loss of proper cell-cell interaction/connection, which compromises the native follicular structure (Woodruff and Shea, 2011; Green and Shikanov, 2016). In the 2D culture condition, despite granulosa cells expansion is faster due to their attached growth, the oocyte survival rate from PGCs is compromised when compared to that in the 3D system. Therefore, the 2D system **is** mainly used for culture of secondary or antral follicles (Lee et al., 2018).

On the contrary, the 3D system is mainly conducted in suspension culture or with the follicles encapsulated within biomaterials mimicking the *in-vivo* conditions. The biomaterials include alginate (Heise et al., 2005; Laronda et al., 2014; Rajabi et al., 2018), collagen (Joo et al., 2016; Rajabi et al., 2018; Yeung et al., 2019), hydrogel (Xu et al., 2009; Shikanov et al., 2011), Matrigel (Sadr et al., 2018) and fibrin clot (Sadr et al., 2018; Kim et al., 2020). Recently, new materials like tyramine-linked hyaluronan are also used (Desai et al., 2022). The 3D system is more suitable for long-term culture. It enhances maturation of the cultured follicles to a stage capable of ovulation and fertilization of the derived oocytes (Desai et al., 2022).

An ovarian organoid formation protocol was lately reported with the use of both 2D and 3D conditions. The *in-vivo* isolated PGCs or stem cell-derived PGCLCs were first mixed with *in-vivo* gonadal somatic cells or stem cell-derived FOSLCs to form aggregates, which were encapsulated in Matrigel or directly cultured in suspension and transferred to collagen-coated inserts for differentiation and growth (Li et al., 2021b; Yoshino et al., 2021). Under such conditions, follicle-like structures and competent oocytes were observed.

The compositions of follicle culture medium vary widely depending on the stage of the cultured follicles. As only the secondary follicles are responsive to reproductive hormones, the culture medium compositions are categorized for *in-vitro* differentiation, activation, growth and maturation. The differentiation medium is established mainly for stem cell-derived ovarian organoids. Special basal medium, commercial α-MEM and stem-pro-34 medium are reported to enhance the initial follicular assembly and growth (Li et al., 2021b; Yoshino et al., 2021). For activation, components such as PI3K pathway promoter, glucose (AMPK/mTOR pathway activator), phosphatidic acid (PA) and propranolol (mTOR pathway stimulators) shown to enhance follicle activation are included. Follistim is commonly used to mimic the hormone-dependent environment, which together with stimulants like BMP15 and GDF9 (Hayashi et al., 2017) enhance *in-vitro* growth and maturation of the cultured follicles. Until now, in human, the *in-vitro* follicles generated solely from PSCs only resembled the primordial follicle-like structures. Whether the follicles have the capacity to develop into more advanced stages requires further investigations to build the foundation for future application.

## 10 Future perspective and conclusion

Over the past decade, huge advance has been achieved on our understanding of folliculogenesis due to emerging techniques such as single-cell RNA sequencing. Successful derivation of PGCs and subsequent oogenesis solely from PSCs has provided insights into *in-vitro* gonadal formation. The understanding paves the way to solve infertility caused by congenital diseases such as turner syndrome (Muntaj et al., 2015; de Souza et al., 2021). It is feasible to produce PGCs from patient derived iPSCs (Yango et al., 2013; de Souza et al., 2021). Moreover, the establishment of disease models offers a chance to understand the mechanism of infertility, and to provide a platform for drug screening and potential therapeutic approaches. Although the granulosa cell differentiation protocol from PSCs remains to be optimized, the current progress and understanding of granulosa cell development is a big step forward for future approaches in rescuing ovarian function in terms of endocrine secretion. *In vitro* developed endocrine cells not only help to understand the aetiology of the diseases such as premature ovarian insufficiency and polycystic ovarian syndrome but brings novel thoughts on possiblility of cell therapy.
